# Involvement of HMGB1 in Resistance to Tumor Vessel-Targeted, Monoclonal Antibody-Based Immunotherapy

**DOI:** 10.1155/2016/3142365

**Published:** 2016-01-27

**Authors:** Vito Pistoia, Annalisa Pezzolo

**Affiliations:** Laboratorio di Oncologia, Istituto Giannina Gaslini, 16147 Genova, Italy

## Abstract

High mobility group box 1 (HMGB1) is a member of the “danger associated molecular patterns” (DAMPs) than can localize in various compartments of the cell (from the nucleus to the cell surface) and subserve different functions accordingly. HMGB1 is implicated in maintenance of genomic stability, autophagy, immune regulation, and tumor growth. HMGB1-induced autophagy promotes tumor resistance to chemotherapy, as shown in different models of malignancy, for example, osteosarcoma, leukemia, and gastric cancer. To the best of our knowledge, there is virtually no information on the relationships between HMGB1 and resistance to immunotherapy. A recent study from our group has shed new light on this latter issue. We have demonstrated that targeting of tumor-derived endothelial cells with an anti-human CD31 monoclonal antibody in a human neuroblastoma model was unsuccessful due to a complex chain of events involving the participation of HMGB1. These results are discussed in detail since they provide the first evidence for a role of HMGB1 in resistance of tumor cells to monoclonal antibody-based immunotherapy.

## 1. Introduction

High mobility group box 1 (HMGB1) is the best characterized member of the so-called “danger associated molecular patterns” (DAMPs), a heterogenous group of molecules that can be derived from any compartment of the cell and are released or secreted by stressed or dead/dying cells in response to sterile inflammation (e.g., trauma, ischemia, autoimmunity, and cancer). DAMPs released in the extracellular milieu alert the immune system of a dangerous situation with the final aim of reestablishing homeostasis. Thus, intracellular DAMPs perform their physiological functions and cannot be detected by the immune system, while extracellular DAMPs act as danger sensors and immunostimulatory molecules [1, 2].

HMGB1 is a highly conserved molecule, present in almost all metazoans and plants. The HMGB1 protein is composed of 215 amino acid residues and is organized in three different domains: (i) A box and B box, two tandem domains, and (ii) a 30-amino acid-long acidic tail in the C-terminal portion of the molecule. HMGB1 box domains bind to DNA in chromatin through protein-protein interactions or recognition of DNA structures. The B box triggers secretion of proinflammatory cytokines by macrophages and this function is competitively blocked by the A box [1, 2]. HMGB1 contains three cysteines: C23 and C45 that can form a disulfide bond and C106 that is unpaired. These cysteine residues are modified by redox reactions that generate three isoforms named “fully reduced HMGB1” for the all-thiol form, “disulfide HMGB1” for the partially oxidized form, and “sulfonyl HMGB1” for the terminally oxidized form [3].

HMGB1 can localize in the nucleus and the cytoplasm and at the cell surface, besides being released extracellularly in a truncated soluble form. Nuclear HMGB1 participates in DNA replication, recombination, transcription, and repair and maintains telomere homeostasis and genomic stability [1, 2, 4]. Under stress conditions, HMGB1 translocates from the nucleus to the cytoplasm where it binds to Beclin-1 (BCN-1) and promotes autophagy (see below), while inhibiting apoptosis [5]. Cell surface HMGB1 promotes neurite outgrowth and platelet activation. Extracellular HMGB1 binds with high affinity to different receptors including the receptor for advanced glycation end products (RAGE), Toll-like receptors (TLRs) 2, 4, and 9, syndecan-1 (CD138), CD24, and T-cell immunoglobulin mucin-3 (Tim-3) [1, 2, 4]. Notably, CD24 [6] and Tim-3 [7] are negative regulators of HMGB1 effects on macrophages and tumor-associated dendritic cells (DCs). The signal transduction pathways activated by soluble HMGB1 include NF-*κ*B, interferon regulatory factor-3 (IRF-3), and phosphoinositide-3-kinase (PI3K) and culminate into immune cell activation, induction of proinflammatory cytokines and type I IFN, stimulation of cell proliferation, angiogenesis, cell adhesion and migration, and autophagy [1, 2]. Interaction of HMGB1 with RAGE is involved in cell migration, either directly by inducing expression of adhesion molecules such as VCAM-1 and ICAM-1 or indirectly by stimulating secretion of chemokines [1, 2, 4], especially CXCL12 which can form a heterocomplex with HMGB1 endowed with potentiated chemotactic activity [8]. Other partners form heterocomplexes with HMGB1 as interleukin- (IL-) 1, DNA, nucleosome or lipopolysaccharide (LPS), with the eventual result of synergistic proinflammatory and immune activities [1, 2, 4]. The biological functions of HMGB1 vary with the redox states of the cysteine residues; thus, the fully reduced isoform has chemokine activity only, the disulfide isoform shows cytokine activity only, and the all oxidized-isoform is devoid of chemokine or cytokine activity [3].

## 2. HMGB1 and Cancer

The role of nuclear HMGB1 in DNA repair and maintenance of genomic stability represents by itself a powerful antitumor mechanism [1, 2].

Immunogenic cell death (ICD) is characterized by the release or the exposure on the surface of dying cells of DAMPs that enhance DC differentiation and antigen uptake and presentation, resulting in potent activation of cytotoxic T lymphocyte responses. ICD contributes to tumor eradication in the course of chemotherapy (e.g., anthracyclines, platinum-based compounds) or radiotherapy. Dying tumor cells expose calreticulin on the cell surface and release ATP in the TME; ATP, in turn, binds to the purinergic receptor P2X7 and activates the NLRP3 inflammasome that promotes IL-1*β* maturation and secretion [9, 10]. Extracellular HMGB1 contributes to chemotherapy-induced ICD by binding to TLR4 on DCs and potentiating antitumor immune responses [11]. However, the overall effects of HMGB1 on tumor growth are complex since, as detailed below, HMGB1 contributes to stimulating neoplastic cell growth and metastasis through different mechanisms, some of which are also involved in the enhancement of antitumor immunity.

The inflammatory tumor microenvironment (TME) induces HMGB1 release by infiltrating leukocytes and the cancer cells themselves. Extracellular HMGB1 in turn binds to RAGE and TLR4 and activates proinflammatory signaling pathways such as NF-*κ*B and inflammasome, thus accelerating tumor growth and metastasis [1, 2, 4, 12]. In pancreatic adenocarcinoma, extracellular HMGB1 has been shown to enhance mitochondrial RAGE expression and translocation, resulting in increased mitochondrial complex I activity and ATP production [13]. These findings, which establish a link between HMGB1 and energy metabolism, are supported by reduced tumorigenesis and ATP production in the TME in RAGE deficient mice [14].

HMGB1 released in the TME induces expression of proangiogenic factors such as vascular endothelial growth factor (VEGF) and their receptors in tumor endothelial cells (EC) through RAGE mediated NF-*κ*B signaling. HMGB1 promotes EC migration and sprouting and sustains a proangiogenic regulatory loop, whereby stimulation of EC with recombinant (r)HMGB1 induces a positive autocrine circuit leading to induction of RAGE and TLR4, as well as of endogenous HMGB1, expression. Therapeutic targeting of HMGB1 inhibits angiogenesis in this model [15].

HMGB1 dampens antitumor immunity by inducing apoptosis of macrophage-derived DCs and suppressing tumor specific CD8^+^ T cell effectors in part through the induction of IL-10 production by T regulatory cells. Finally, HMGB1 promotes tumor infiltration by lymphotoxin *α*1*β*2-producing T cells which recruit M2-type macrophages that support tumor angiogenesis and growth [4].

**Figure 1:**
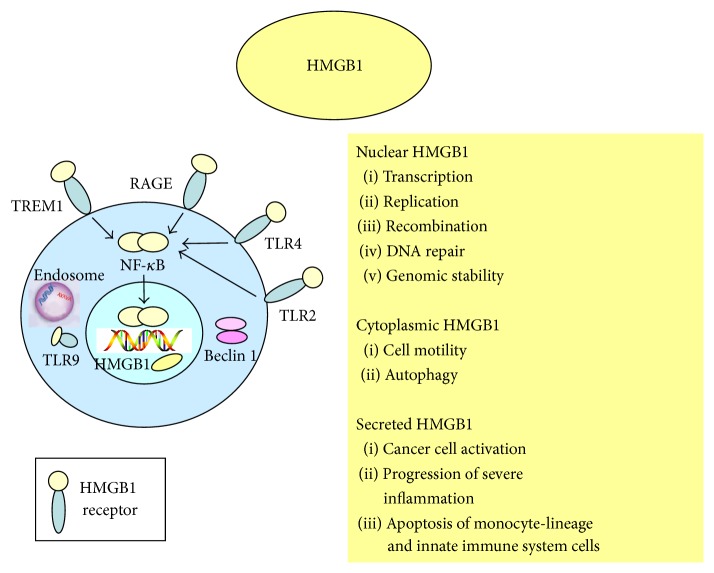
Functions of high mobility group protein B1 (HMGB1). HMGB1 modulates inflammation, immunity, chemotaxis, and tissue regeneration. HMGB1 leads to NF-*κ*B activation that in turn can upregulate inflammatory cytokine production, HMGB1 secretion, and HMGB1 receptor expression (TRL2, TRL24, RAGE, TRL9, and TREM1). HMGB1 participates in DNA replication, recombination, transcription, and repair. HMGB1 interacts with TLR9 in the endoplasmic reticulum-Golgi intermediate compartment. HMGB1 promotes autophagy. Cytosolic HMGB1 binds to Beclin-1 which promotes autophagosome formation and dynamic intracellular trafficking during autophagy.

Autophagy is a programmed process of cell survival based upon lysosome-mediated degradation of cell components (e.g., damaged organelles) and of invading pathogens in a selective or a nonselective modality. Autophagy is primarily controlled by the autophagy-related (ATG) protein family including ATG5, ATG7, and Beclin- (BECN-) 1 but can also occur in an ATG pathway-independent manner [16]. HMGB1 intervenes in the autophagic process at various levels. Nuclear HMGB1 modulates the expression of heat shock protein (HSP) *β*1 through a pathway that requires phosphorylation of the latter protein at residues Ser15 and Ser86. HSP*β*1 is a regulator of the cytoskeleton that controls intracellular trafficking during autophagy and mitophagy, that is, the selective degradation of damaged mitochondria. Inhibition of the HMGB1-HSP*β*1 pathway results in deficiency of both autophagy and mitophagy. Cytoplasmic HMGB1 interacts directly with BECN-1 and dissociates it from Bcl-2; the successful accomplishment of this process requires the disulfide HMGB1 isoform. The unc-21-like kinase positively regulates this type of HMGB1-mediated autophagy, whereas TP53 inhibits it. Extracellular reduced HMGB1 induces autophagy and tumor growth through RAGE, whereas oxidized HMGB1 triggers apoptosis of cancer cells [1, 2, 4, 5].  Figure 1 summarizes the main biologic activity of nuclear, cytoplasmic, and secreted HMGB1.

It is now clear that HMGB1 induced autophagy promotes tumor resistance to chemotherapy. This has been shown in different models of malignancy such as osteosarcoma, leukemia, and gastric cancer [17–19]. In contrast, scarce information is available on the relationships between HMGB1 and resistance to immunotherapy. A recent study from our group that is here discussed has shed new light on this latter issue.

## 3. HMGB1 and Neuroblastoma

Neuroblastoma (NB) is a pediatric malignancy originating from the neural crest that presents with metastatic disease at diagnosis in approximately a half of patients (high risk cases). According to the American Cancer Society (http://www.cancer.org/cancer/neuroblastoma) the 5-year survival of NB patients in the low risk group is higher than 95%, in the intermediate risk group is approximately 90%, and in the high risk group is around 40% to 50%. A therapeutic protocol based upon the combination of anti-GD2 antibody, GM-CSF, IL-2, and isotretinoin has shown great efficacy in a cohort of high risk NB patients, with 2-year event-free survival of 66% and overall survival of 86% [20]. These figures have been recently updated to 74% and 84%, respectively [21].

As in other malignancies, angiogenesis is a major determinant of NB growth and progression. Some years ago, we discovered that variable proportions of NB microvessels were lined by tumor-derived endothelial cells (TDEC), both in tumors formed by the human NB cell line HTLA-230 in immunodeficient mice and in primary tumors (3/10 cases tested). In particular, approximately 50% of the microvessels in experimental tumors and 20–80% in primary NB masses were of tumor origin, as demonstrated by EC expression of classical endothelial markers (CD31, CD105, and von Willebrand factor) and amplification of the MYCN oncogene [22, 23]. Subsequent studies from our group demonstrated that these NB cells disguised as EC and lining microvessels in the TME expressed also typical neuroblastic markers such as GD2, CD56, and NB-84 [24]. Finally, tumor-derived endothelial microvessels were coated by host derived pericytes that never showed MYCN amplification [23].

These results provided an unique opportunity to investigate whether selective targeting of TDEC in our NB orthotopic model closely mimicking primary NB development impacted on tumor growth and mouse survival. TDEC have been implicated in resistance to chemotherapy and tumor progression [25]. Thus, SCID/NOD mice bearing tumors formed by the HTLA-230 cell line were treated with the anti-human (h)CD31 monoclonal antibody (mAb) Moon-1 or with PBS as control [26]. CD31 is a member of the immunoglobulin gene superfamily expressed on the surface of ECs, as well as of various hematopoietic cells including platelets, neutrophils, monocytes, megakaryocytes, natural killer cells, and T and B cells. Numerous heterophilic ligands of CD31 have been identified including the *α*v*β*3 integrin, the CD38 ectoenzyme, and CD177 expressed on a subset of neutrophils [27, 28]. The* in vivo* relevance of these CD31 heterophilic ligands is unknown. In ECs, CD31 localizes to the borders of adjacent cells and mediates leukocyte migration through ECs and the EC basement membrane. CD31 mediates both outside-in and inside-out signaling. The former are initiated by CD31 ligation and dimerisation and the latter is initiated by integrin ligation, shear stress, cytokines, and other stimuli. The intracellular portion of CD31 contains two immunoreceptor inhibitory motifs that serve as docking sites for signaling molecules such as protein tyrosine phosphatases, whose binding induces phosphorylation of tyrosine- and serine/threonine residues. This latter event, in turn, promotes recruitment of SH2-containing phosphatases (SHP-1/2 and SHIP) and phospholipase C-*γ*1, eventually culminating in cell activation [27, 28].

The CD31 mAb Moon-1 does not react with mouse CD31 and therefore targets human EC only, including those derived from NB cells. Survival of mice treated with the Moon-1 mAb or PBS was comparable, indicating the inefficacy of hCD31-targeted immunotherapy. Immunohistological analyses of tumor masses showed that TDEC apoptosis was significantly higher in mAb-treated than control mice, consistent with the cytotoxic activity of Moon-1. Concomitantly, a significant increase of Ki67^+^, proliferating EC was detected in mAb-treated mice, suggesting the occurrence of vascular remodelling following hCD31 mAb administration whereby TDEC killed by the latter mAb were rapidly replaced by new ones rapidly differentiated from NB cells. In addition, mouse EC were increased after mAb treatment [29].

To gain more insight into the mechanisms underlying such remodelling we first performed gene expression analysis of tumors from treated and control mice, focusing on human angiogenesis-related genes. hCD31 mAb treatment induced outstanding upregulation of the expression of numerous proangiogenic genes including CCL11, CXCL3, CXCL5, cadherin 5, also known as vascular endothelial (VE) cadherin, collagen type IV *α*3, vascular endothelial growth factor, platelet-derived growth factor-A, fibroblast growth factor-1, tumor necrosis factor, and interleukin-6. In contrast, expression of mouse proangiogenic genes was not modulated by mAb immunotherapy [29]. All of these human proangiogenic factors, that are non-species-specific, likely stimulated both tumor-derived and mouse EC regeneration following TDEC depletion by hCD31 mAb. We also identified a small subpopulation of NB cells that express tenascin-C (TNC) on the cell surface and the stem cell transcription factor Oct-4, which serve as progenitors of TDEC. These cells were found to be increased following hCD31 mAb treatment, consistent with the hypothesis formulated above [29].

**Figure 2:**
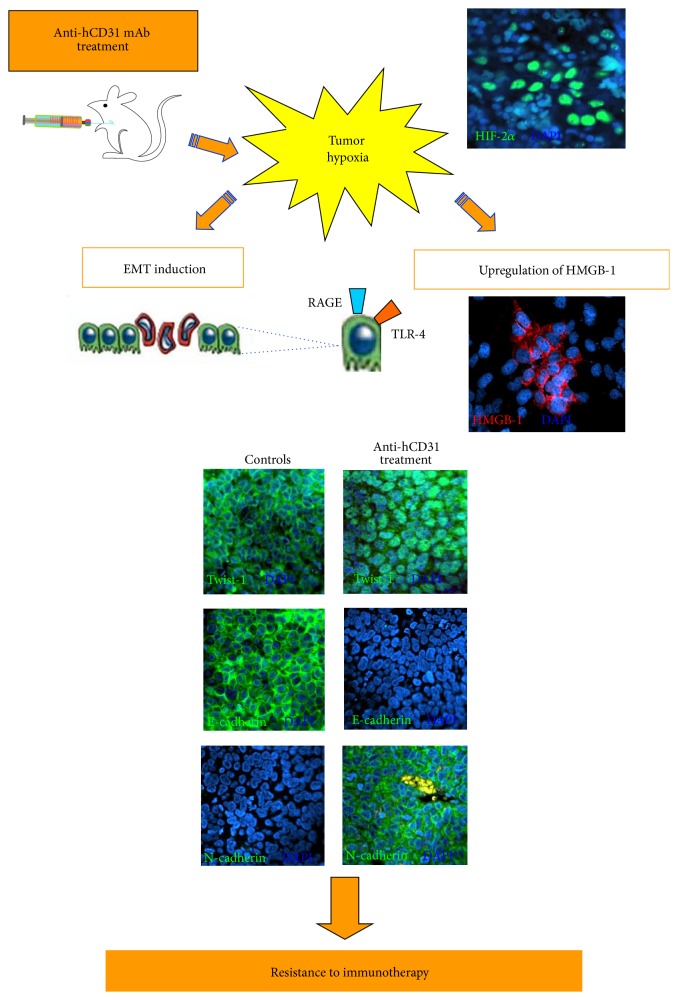
A model for HMGB1 involvement in resistance to mAb-based immunotherapy. hCD31 mAb-driven hypoxia promotes in NB cells expression of HMGB1 that induces by itself EMT, thus mimicking the effects of hypoxia and serving as an amplification loop. All of these mechanisms in combination account for the refractoriness of NB tumors to TDEC targeting with hCD31 mAb.

TNC is a multifunctional protein of the extracellular matrix that binds to HMGB1 and is involved in epithelial to mesenchymal transition (EMT) [30]. Thus, we investigated the expression of a panel of EMT-related genes in the same tumor samples tested for angiogenesis-related gene expression. EMT-related transcripts, including epidermal growth factor, hepatocyte growth factor, insulin growth factor-1, tumor necrosis factor, CXCL5, IL-6, fibroblast growth factor-1, platelet-derived growth factor-A, and matrix metalloprotease 2, were found to be upregulated in tumors from hCD31 mAb treated* versus* control mice. The next step was to test the expression of few EMT-related proteins, as well as of HMGB1, in tumor tissue sections from hCD31 treated and control mice. It was found that Twist-1, a master regulator of EMT [30], displayed cytoplasmic localization, consistent with a transcriptional inactive state, in tumors from control mice, whereas it was detected in the nucleus of most malignant cells, indicative of ongoing transcriptional activity, in tumors from hCD31 treated mice (Figure 2). The latter tumors lost expression of E-cadherin and acquired expression of N-cadherin, two typical features of EMT, whereas opposite patterns were detected in control tumors (Figure 2). Finally, HMGB1 was always found in cytoplasmic location, but the proportion of HMGB1 cells increased very significantly in tumors from hCD31 mice* versus* controls [29]. Taken together, these studies demonstrated that EMT was involved in the failure of hCD31 mAb treatment and that HMGB1 was an additional player in this phenomenon (Figure 2).

Hypoxia promotes translocation of HMGB1 from the nucleus to the cytoplasm and increases expression of RAGE in the TME [31, 32]. We reasoned that TDEC depletion caused by hCD31 mAb treatment might reduce blood supply and increase tumor hypoxia. Since hypoxia induced factor 2*α* (HIF2*α*) is upregulated in NB cells in hypoxic conditions [33] and has been associated with developing endothelium [34], we stained for HIF2*α* from tumor sections from mice treated with hCD31 mAb or control mice (Figure 1). The proportion of HIF2*α* ^+^ cells was significantly higher in the former than in the latter tumors, indicating that (i) tumor-derived microvessels were fully functional, (ii) hypoxia was enhanced by selective targeting of TDEC, and (iii) the latter condition was likely involved in HMGB1 upregulation following hCD31 treatment [29]. Hypoxia is also an inducer of EMT [35], so we were interested in investigating whether the results obtained* in vivo* could be replicated* in vitro*. Indeed, human NB cell lines cultured under hypoxic conditions displayed Twist-1 nuclear localization, acquisition of N cadherin, and loss of E cadherin expression (EMT features) compared to the same cells maintained in normoxia. Furthermore, hypoxia induced the appearance of endothelial cell markers (CD31, VE cadherin) and upregulation of cytoplasmic HMGB1 in NB cell lines [29]. Thus, both* in vivo* and* in vitro* data point to hypoxia as a common inducer of EMT and transdifferentiation of NB progenitor cells into TDEC.

HMGB1 induces EMT in lung and renal fibrosis [36, 37], so we asked whether HMGB1 could mimic the effects of hypoxia on human NB cells. To answer this question, the latter cells were cultured with rHMGB1 or medium alone in normoxic conditions and tested for the expression of TNC, as marker of EMT, and VE cadherin and CD31, as markers of TDEC. rHMGB1 induced the expression of all these markers, suggesting that HMGB1 itself was an inducer of EMT and TDEC differentiation in normoxia and recapitulated at least in part the effects of hypoxia on NB cells [29].

A careful review of the recent literature has not disclosed any paper addressing specifically the role of HMGB1 in resistance to immunotherapy. Interestingly, however, it has been shown that cancer-associated fibroblasts that are strongly involved in tumor progression induced HMGB1 upregulation in breast cancer cells, thus contributing to resistance of the latter cells to doxorubicin [38]. Although in our study tumor cells appeared to be the major source of HMGB1, the possibility that stromal cells as cancer-associated fibroblasts contributed to its production cannot be excluded. Another mechanism of tumor resistance to chemotherapy that may apply also to escape from immunotherapy has been recently identified. It has been shown that overexpression of micro(mi)RNA-218 sensitized paclitaxel resistant endometrial carcinoma cells to paclitaxel by binding to the 3′-UTR of the HMGB1 gene, with downregulation of HMGB1 expression and suppression of HMGB1-mediated autophagy [39]. Another recent study demonstrated that HMGB1 released from dying cells induced secretory/cytoplasmic clusterin in prostate cancer cells [40]. This latter molecule is a potent antiapoptotic protein that binds to and sequesters Bax from mitochondria, thus preventing caspase 3 activation [41]. HMGB1 induced clusterin protected prostate cancer cells from docetaxel, an antitumor drug [40]. Once again, similar mechanisms might operate in case of resistance of cancer cells to immunotherapy.

## 4. Conclusions

HMGB1 is involved in resistance to mAb-based immunotherapy in an experimental model where the target antigen is expressed by TDEC. The mechanisms of resistance are numerous and initiated by hypoxia that is a common condition of the TME but was here increased by hCD31 mAb operated depletion of TDEC. Hypoxia, in turn, was the driver of upregulation of EMT-related gene expression, up-regulation of vascular mimicry-related genes (not discussed here), and induction of the expression of cytoplasmic HMGB1, consistent with the chemotactic activity of the latter molecule. All of these events that were closely interconnected converged upon stimulation of differentiation of TNC^+^, Oct4^+^ NB progenitor cells to TDEC, and generation of novel tumor-derived endothelial microvessels, which thwarted the activity of hCD31 mAb (Figure 2). The model proposed to explain the failure of CD31 targeting in our experimental conditions does not take into account the potential involvement of additional players, such as stromal cells or other constituents of the TME.

Unexpectedly, rHMGB1, an equivalent of endogenous extracellular HMGB1, mimicked some of the effects of hypoxia on NB cells, namely, induction of EMT and differentiation to TDEC. We speculated that HMGB1 released in the TME might potentiate the effects of hypoxia in less hypoxic areas of the tumor. Further studies are needed to broaden our knowledge of the role of HMGB1 in resistance to mAb and cellular immunotherapy.

## References

[1] Sims G. P., Rowe D. C., Rietdijk S. T., Herbst R., Coyle A. J. (2010). HMGB1 and RAGE in inflammation and cancer. *Annual Review of Immunology*.

[2] Andersson U., Tracey K. J. (2011). HMGB1 is a therapeutic target for sterile inflammation and infection. *Annual Review of Immunology*.

[3] Venereau E., Casalgrandi M., Schiraldi M. (2012). Mutually exclusive redox forms of HMGB1 promote cell recruitment or proinflammatory cytokine release. *The Journal of Experimental Medicine*.

[4] Kang R., Zhang Q., Zeh H. J., Lotze M. T., Tang D. (2013). HMGB1 in cancer: good, bad or both?. *Clinical Cancer Research*.

[5] Sun X., Tang D. (2014). HMGB1-dependent and -independent autophagy. *Autophagy*.

[6] Chen G.-Y., Tang J., Zheng P., Liu Y. (2009). CD24 and siglec-10 selectively repress tissue damage—induced immune responses. *Science*.

[7] Chiba S., Baghdadi M., Akiba H. (2012). Tumor-infiltrating DCs suppress nucleic acid–mediated innate immune responses through interactions between the receptor TIM-3 and the alarmin HMGB1. *Nature Immunology*.

[8] Schiraldi M., Raucci A., Muñoz L. M. (2012). HMGB1 promotes recruitment of inflammatory cells to damaged tissues by forming a complex with CXCL12 and signaling via CXCR4. *The Journal of Experimental Medicine*.

[9] Obeid M., Tesniere A., Ghiringhelli F. (2007). Calreticulin exposure dictates the immunogenicity of cancer cell death. *Nature Medicine*.

[10] Casares N., Pequignot M. O., Tesniere A. (2005). Caspase-dependent immunogenicity of doxorubicin-induced tumor cell death. *The Journal of Experimental Medicine*.

[11] Apetoh L., Ghiringhelli F., Tesniere A. (2007). Toll-like receptor 4-dependent contribution of the immune system to anticancer chemotherapy and radiotherapy. *Nature Medicine*.

[12] Castellani P., Balza E., Rubartelli A. (2014). Inflammation, DAMPs, tumor development, and progression: a vicious circle orchestrated by redox signaling. *Antioxidants & Redox Signaling*.

[13] Kang R., Tang D., Schapiro N. E. (2013). The HMGB1/RAGE inflammatory pathway promotes pancreatic tumor growth by regulating mitochondrial bioenergetics. *Oncogene*.

[14] Gebhardt C., Riehl A., Durchdewald M. (2008). RAGE signaling sustains inflammation and promotes tumor development. *Journal of Experimental Medicine*.

[15] van Beijnum J. R., Nowak-Sliwinska P., van den Boezem E., Hautvast P., Buurman W. A., Griffioen A. W. (2013). Tumor angiogenesis is enforced by autocrine regulation of high-mobility group box 1. *Oncogene*.

[16] Mizushima N. (2007). Autophagy: process and function. *Genes & Development*.

[17] Huang J., Ni J., Liu K. (2012). HMGB1 promotes drug resistance in osteosarcoma. *Cancer Research*.

[18] Liu L., Yang M., Kang R. (2011). HMGB1-induced autophagy promotes chemotherapy resistance in leukemia cells. *Leukemia*.

[19] Zhan Z., Li Q., Wu P. (2012). Autophagy-mediated HMGB1 release antagonizes apoptosis of gastric cancer cells induced by vincristine via transcriptional regulation of Mcl-1. *Autophagy*.

[20] Yu A. L., Gilman A. L., Ozkaynak M. F. (2010). Anti-GD2 antibody with GM-CSF, interleukin-2, and isotretinoin for neuroblastoma. *The New England Journal of Medicine*.

[21] Ozkaynak M., Gilman A. L., Yu A. L. (2014). A comprehensive safety trial of chimeric antibody 14. 18 (ch14. 18) with GM-CSF, IL-2 and isotretinoin in high-risk neuroblastoma patients following myeloablative therapy: a children's oncology group study. *Advances in Neuroblastoma Research*.

[22] Ribatti D., Nico B., Pezzolo A. (2006). Angiogenesis in a human neuroblastoma xenograft model: mechanisms and inhibition by tumour-derived interferon-*γ*. *British Journal of Cancer*.

[23] Pezzolo A., Parodi F., Corrias M. V., Cinti R., Gambini C., Pistoia V. (2007). Tumor origin of endothelial cells in human neuroblastoma. *Journal of Clinical Oncology*.

[24] Pezzolo A., Parodi F., Marimpietri D. (2011). Oct-4^+^/Tenascin C^+^ neuroblastoma cells serve as progenitors of tumor-derived endothelial cells. *Cell Research*.

[25] McGuire T. F., Sajithlal G. B., Lu J., Nicholls R. D., Prochownik E. V. (2012). In vivo evolution of tumor-derived endothelial cells. *PLoS ONE*.

[26] Deaglio S., Morra M., Mallone R. (1998). Human CD38 (ADP-ribosyl cyclase) is a counter-receptor of CD31, an Ig superfamily member. *The Journal of Immunology*.

[27] Ilan N., Madri J. A. (2003). PECAM-1: old friend, new partners. *Current Opinion in Cell Biology*.

[28] Woodfin A., Voisin M.-B., Nourshargh S. (2007). PECAM-1: a multi-functional molecule in inflammation and vascular biology. *Arteriosclerosis, Thrombosis, and Vascular Biology*.

[29] Pezzolo A., Marimpietri D., Raffaghello L. (2014). Failure of anti tumor-derived endothelial cell immunotherapy depends on augmentation of tumor hypoxia. *Oncotarget*.

[30] Martin A., Cano A. (2010). Tumorigenesis: twist1 links EMT to self-renewal. *Nature Cell Biology*.

[31] Yan W., Chang Y., Liang X. (2012). High-mobility group box 1 activates caspase-1 and promotes hepatocellular carcinoma invasiveness and metastases. *Hepatology*.

[32] Tafani M., Schito L., Pellegrini L. (2011). Hypoxia-increased RAGE and P2X7R expression regulates tumor cell invasion through phosphorylation of Erk1/2 and Akt and nuclear translocation of NF-*κ*B. *Carcinogenesis*.

[33] Mohlin S., Hamidian A., Pahlman S. (2013). HIF2*α* and IGF2 expression correlates in human neuroblastoma and normal immature sympathetic neuroblasts. *Neoplasia*.

[34] Paulis Y. W. J., Soetekouw P. M. M. B., Verheul H. M. W., Tjan-Heijnen V. C. G., Griffioen A. W. (2010). Signalling pathways in vasculogenic mimicry. *Biochimica et Biophysica Acta—Reviews on Cancer*.

[35] Kalluri R., Weinberg R. A. (2009). The basics of epithelial-mesenchymal transition. *Journal of Clinical Investigation*.

[36] He M., Kubo H., Ishizawa K. (2007). The role of the receptor for advanced glycation end-products in lung fibrosis. *American Journal of Physiology—Lung Cellular and Molecular Physiology*.

[37] Lynch J., Nolan S., Slattery C., Feighery R., Ryan M. P., McMorrow T. (2010). High-mobility group box protein 1: a novel mediator of inflammatory-induced renal epithelial-mesenchymal transition. *American Journal of Nephrology*.

[38] Amornsupak K., Insawang T., Thuwajit P., O-Charoenrat P., Eccles S. A., Thuwajit C. (2014). Cancer-associated fibroblasts induce high mobility group box 1 and contribute to resistance to doxorubicin in breast cancer cells. *BMC Cancer*.

[39] Ran X., Yang J., Liu C., Zhou P., Xiao L., Zhang K. (2015). MiR-218 inhibits HMGB1-mediated autophagy in endometrial carcinoma cells during chemotherapy. *International Journal Clinical Experimental Pathology*.

[40] Zhou J., Chen X., Gilvary D. L. (2015). HMGB1 induction of clusterin creates a chemoresistant niche in human prostate tumor cells. *Scientific Reports*.

[41] Djeu J. Y., Wei S. (2009). Clusterin and chemoresistance. *Advances in Cancer Research*.

